# Social marketing including financial incentive programs at worksite cafeterias for preventing obesity: a systematic review

**DOI:** 10.1186/s13643-019-0965-0

**Published:** 2019-02-28

**Authors:** Kimi Sawada, Koji Wada, Sadequa Shahrook, Erika Ota, Yukari Takemi, Rintaro Mori

**Affiliations:** 1 Department of Food Science and Nutrition, Faculty of Human Life and Environmental Sciences, Nagoya Women’s University, 3-40 Shioji-cho, Mizuho-ku, Nagoya-shi, Aichi 467-8610 Japan; 2 Department of Health Policy, National Center for Child Health and Development, 2-10-1 Okura, Setagaya-ku, Tokyo, 157-8535 Japan; 3 The International University of Health and Welfare, 1-24-1 Minamiaoyama, Minato-ku, Tokyo, 107-0062 Japan; 4 Population Health Research Institute, Global Health, McMaster University and Hamilton Health Sciences, 20 Copeland Avenue, Hamilton, Ontario L8L 0A3 Canada; 5 Global Health Nursing, St. Luke’s International University Graduate School of Nursing Science, 10-1 Akashicho, Chuo-ku, Tokyo, 104-0044 Japan; 6 Nutrition Ecology Department of Nutrition Sciences, Kagawa Nutrition University (Joshi Eiyo Daigaku), 3-9-21 Chiyoda, Sakado, Saitama, 350-0288 Japan

**Keywords:** Incentive-based, Food environmental interventions, Obesity, Systematic review, Workplace

## Abstract

**Background:**

As with food-taxation strategies, such interventions as discounted healthy menus, point-of-purchase advertisements, and sugar-free beverages for employees at worksites could help prevent obesity. This study assessed the effectiveness of food environment interventions incorporating financial incentive or social marketing strategies at workplace cafeterias, vending machines, and kiosks toward preventing obesity and improving dietary habits.

**Methods:**

We conducted searches on CENTRAL, MEDLINE, EMBASE, CINAHL, and PsycINFO databases. The study designs included were randomized control trials (RCTs) and cluster RCTs. We evaluated the effectiveness of financial incentive or social marketing strategies interventions (such as discounts) on health outcomes or food intake behavior. Two reviewers independently screened the studies for inclusion. We assessed the risk of bias using the Cochrane Collaboration’s tool. This protocol was published in 2014.

**Results:**

We included three trials, with a combined total of 3013 participants. There were limited available data from RCTs on changes in body weight. No eligible social marketing studies were retrieved. In some cases, a meta-analysis could not be conducted owing to differences in the analytic methods for the outcomes.

**Conclusions:**

Lack of evidence made it difficult to draw any conclusions. In future surveys, it will be necessary to conduct interventions focusing only on financial incentive intervention versus no intervention in order to determine whether the incentive strategy has a clear impact.

**Systematic review registration:**

PROSPERO CRD4201401056

**Electronic supplementary material:**

The online version of this article (10.1186/s13643-019-0965-0) contains supplementary material, which is available to authorized users.

## Background

Obesity is a leading cause of metabolic syndrome and lifestyle-related diseases such as diabetes, high blood pressure, and high cholesterol [1–3]. According to the Organisation for Economic Co-operation and Development (OECD) [4, 5], the proportion of adults who are overweight, including obese adults with a body mass index (BMI) ≧ 30, has been increasing steadily worldwide since 2000. In particular, approximately 30% of the population is obese in the United States (US), Mexico, and New Zealand, and more than one in four adults are obese in Australia (28.3%), Canada (25.8%), and Chile (25.1%) [5, 6].

The World Health Organization (WHO) emphasized that proper diet and nutrition, including an avoidance of excessive intake of calories, saturated fatty acids, and added sugar in food, are crucial to the primary prophylaxis of chronic diseases [7]. Furthermore, consumption of fruit, vegetables, polyunsaturated and monounsaturated lipids, and whole grains has been shown to be associated with a reduced risk of cancer, ischemic stroke, and heart disease [8–12], and WHO recommendations include an increased intake of fruit and vegetables, whole grains, beans, dietary fiber, and nuts [7, 13].

Obesity is most prevalent among 40- to 60-year-olds [14]. As this demographic is highly represented in the workplace, the company cafeteria is an ideal setting for introducing dietary interventions that focus on eating and buying habits, not only for workers in general but also for this high-risk group in particular. People typically spend more than one third of their daily lives in the workplace, and many use employee cafeterias, vending machines, and kiosks more than once a day; thus, the workplace is an important location for promoting changes in the eating behavior of the working generation. A population approach to reducing obesity has the potential to change eating behavior and has a much broader coverage than group interventions [15].

Behavioral science theory combined with psychology, sociology, health education pedagogy, and nutrition education, such as the Health Belief Model [16], the Trans-Theoretical Model [17] or Social Cognitive Theory [18] for individual interventions, and Social Marketing [19] or Innovation Theory [20] for organizing interventions, has been shown to provide effective approaches to engendering behavioral change [21]. Recently, “financial incentive” strategies including food taxation, discounted prices, and points systems or token economies have been used as food environment interventions [19, 20]. Furthermore, social marketing in behavioral science theory comprises four concepts: product, price, place, and promotion. Price refers to the price of a person’s resources (e.g., money, time, effort) [19]. We decided to include “social marketing” and used price, such as discounts, in this intervention program. We defined social marketing as signifying education programs throughout the workplace; however, social marketing usually has a much broader meaning, and it often refers to public education campaigns for all places [22, 23]. In particular, national policies on food taxation, such as the tax in Denmark on foods high in saturated fat [24] as well as the junk food tax in Hungary [25] and sugar-sweetened beverage tax in the United Kingdom [26] targeting diet at the national level to prevent non-communicable diseases, have drawn considerable attention and gained traction through the efforts of the WHO [13]. Several studies have investigated the influence in workplace settings of price discounts on healthy food items at cafeterias, sugar-free beverages, and low-fat snacks from vending machines or kiosks and free servings of fruit and vegetables on food purchasing behavior [27–29]. However, most reviews of these types of intervention do not only focus on food consumption but also evaluate the quality of evidence of physical measurements and blood test findings (e.g., cholesterol) relevant to obesity. These outcomes are important toward preventing obesity and lifestyle-related diseases among workers; thus, a comprehensive, systematic review of them is required.

## Objectives

The purpose of this systematic review was to evaluate the effectiveness on health outcomes or food intake behavior at the population level of financial incentive policies applied to workplace cafeterias, vending machines, or kiosks in preventing obesity among employees.

## Methods

The protocol of this systematic review has been published elsewhere (PROSPERO, CRD4201401056) [15]. In this study, we adhered strictly to a protocol based on the Cochrane Systematic Review method [30]. We included randomized control trials (RCTs) and cluster RCTs and excluded quasi-RCTs and crossover RCTs. Studies were included based on the following criteria: (1) participants were employees at any worksite and included both men and women; (2) intervention types were organization-based, food-based, incentive-pricing strategies or social marketing applied to workplace cafeterias, vending machines, and kiosks. The primary outcomes of the study were changes in weight (kg), body mass index (BMI) (kg/m^2^), and changes in hemoglobin A1c (HbA1c) (%). The secondary outcomes were blood pressure (mmHg), changes in cholesterol levels (mg), food consumption (changes in vegetable consumption [g or serving (SV)], changes in fruit consumption [g or SV], changes in fruit and vegetable consumption [g or SV], changes in the consumption of sugary beverages[g], changes in the consumption of sweets [g] and other foods [g] ), and nutritional intake (changes in fat and oil intake [g], changes in fiber intake [g], and changes in energy intake [kcal]).

### Search strategy

We searched the following scientific databases from the commencement of the study to January 13, 2016, and we conducted an update on November 18, 2017: CENTRAL (Cochrane Central Register of Controlled Trials), MEDLINE, EMBASE, PsycINFO, and CINAHL. We included all languages in our search and also hand-searched conference proceedings and reference lists of all the included studies and review articles.

### Exclusion criteria

Studies were excluded if they (1) were observational, quasi-experimental, or cross-over in design; (2) focused on exercise-based interventions or on interventions that did not incorporate incentive-based pricing strategies, coupons, free food, or social marketing or included individualized education programs; and (3) included participants who were unemployed, retired or not working, or included only pregnant women, people with allergies, or serious physical or mental illnesses.

### Data collection and assessment of quality of studies

Two reviewers (KS and KW) independently screened the titles and abstracts to find eligible studies. After excluding studies that did not clearly meet the inclusion criteria, we collected the full text of the remaining studies. KS and KW independently assessed these studies against the eligibility criteria using a data extraction form adapted from the *Cochrane Collaboration Handbook* [30]. The same reviewers also independently assessed the risk of bias in accordance with the Cochrane Collaboration’s risk-of-bias tool, which is composed of seven domains: random sequence generation, allocation concealment, blinding of participants and personnel, blinding of outcome assessment, incomplete outcome data, selective reporting, and other bias [30]. We judged each domain as high, low, or unclear risk of bias. If disagreements occurred between the two reviewers, we consulted with the other authors (SS and EO). Any further disagreement was resolved by discussion among all the authors.

### Data analysis/synthesis

We conducted statistical analysis using the Cochrane Collaboration software (Review Manager Version 5.3) [31]. Continuous variables were evaluated using a random effects model and presented as the average range with a 95% confidence interval (CI), a *p* value of 0.05, and estimates of Tau^2^ and *I*^2^. If the outcomes of the included studies were insufficient, we did not combine the trials. We used the Grades of Recommendation, Assessment, Development and Evaluation Working Group (GRADE) [32] guidelines to assess the quality of evidence for important outcomes. The GRADE approach consists of five categories (limitations or risk of bias, consistency of effect, indirectness of evidence, imprecision, and publication bias) for assessing the quality of evidence for seven or fewer main outcomes. The quality rating of the evidence was presented in four levels (high, moderate, low, very low). Outcomes of the RCT were downgraded from “high quality” by one level for serious limitations (or by two levels for very serious limitations), depending on the assessments of the risk of bias, indirectness of evidence, serious inconsistency, imprecision of effect estimates, or potential publication bias [30]. The GRADE approach was used to assess the quality of the evidence for six major outcomes: weight changes, BMI, HbA1c, blood pressure (BP), cholesterol ( total cholesterol (TC), low-density lipoprotein (LDL), high-density lipoprotein (HDL)), and fruit intake.

## Results

### Description of studies

See Table 1 and Table 2.

**Table 1:** Characteristics of included studies (randomized controlled trials)

Characteristic	Study 1	Study 2	Study 3
Reference no.	33	34	35
Author	Vermeer et al.	Lowe et al.	Thorndike et al.
Year	2011	2010	2016
Country	Netherlands	USA (Philadelphia)	USA (Massachusetts)
Type of study	Cluster RCT	RCT	RCT
Participants	Hospital: *N* = 15, company: *N* = 5, university: *N* = 3, police department: *N* = 2	Hospital: *N* = 2 cafeterias in hospital or university employees	Hospital: *N* = 2 cafeterias in hospital where employees were working
Total study population (I/C)	Pre: 499 (184, 135/180) Post: 308 (129, 75/104)	96 (47/49)	
Sex	Males and females; male 50%	Males and females (18, 78), EC (11, 38), ECPls (7, 40)	Male and females; feedback incentive (72, 28), feedback only (73,27), control (72, 28)
Age	18–79 years; mean (SD) = 39.18 (11.26)	21–65 years	18–50 and over
Intervention duration	3 months	3 months	3 months
Follow-up	–	6 months, 12 months	1 month, 2 months, 3 months
Intervention program	1. Intervention group 1 (*N* = 9): price was 65% of the standard price. About 2/3 of the size of the standard portion was offered. 2. Intervention group 2 (*N* = 8); price was 80% of the standard price. A smaller portion size was added to the assortment and value-size pricing (a lower price per unit for large portions than for small portions).	1. Intervention group (density education and incentive): environmental change (EC)-plus ・Financial discounts: 15% discount (low-energy density) or 25% discount (very low-energy density) for cafeteria food items which were lower in energy density (e.g., soups, salads, diet soda, any entrees or side dishes etc., labeled as low or very low in energy density) *Green: very low in energy density (< 0.6 kcal/g) *Yellow: low in energy density (< 0.6–1.5 kcal/g) *Orange: medium in energy density: (< 1.6–3.9 kcal/g) *Red: high in energy density: (< 4.9–9.0 kcal/g) ・Group sessions (four time × 60 min) during which subjects were informed about the energy density of different food items.	1. Intervention group 1 (feedback incentive): ・Rewards for achieving “green goal” (40%, 60%, 80%) of all cafeteria purchases. Each time the goal was achieved in a month, $10 was earned as a reward. ・Same as feedback-only group
Control program	1. The control group (*N* = 8): the standard size of hot meal was offered.	1. Control group (only environmental changes): EC・(same as EC-plus)・No financial discounts No group session	1. Control group (no contact) 2. Feedback only (four letters sent over a period of 3 months; explanation of traffic light system or the proportion of employee’s traffic light group purchases)
ITT*	No; pre-post	Yes	No; pre-post
Outcome	Primary outcome: BMI Secondary outcome: Fried snacks	Primary outcome: weight change Secondary outcomes: Cholesterol (TC, HDL, LDL), blood pressure (no outcome data), food intake (fruits, meats, dairy, breads, dairy products, fat and sweets), nutritional intake (total energy kcal), sales data (purchased energy [kcal] and purchased proportion of calories from fat, protein, and carbohydrate)	Primary outcome: none Secondary outcomes: inappropriate

*Intention-to-treat test (ITT): Intervention consisting of financial incentive program versus no financial incentive program

*RCT* randomized controlled trial, *I/C* intervention/control, *EC* control group (financial incentive and environmental changes), *EC-plus* intervention group (density education and financial incentive and environmental change), *SD* standard deviation, *BMI* body mass index, *TC* total cholesterol, *HDL* high-density lipoprotein, *LDL* low-density lipoprotein

**Table 2:** Characteristics of excluded studies (randomized controlled trials)

Characteristic	Study 1	Study 2	Study 3
Reference no.	36	37	38, 39
Author	Lachat et al.	French et al.	French et al., French et al.
Year	2009	2001	2010, 2010
Reason for exclusion	Participants were not only employees but also university students	Participants were not only worksite employees but also secondary school students	Interventions included fitness facility environmental interventions or physical activity enhancement like yoga or walking class interventions.
Country	Belgium	USA: Minneapolis-St. Paul, Minneapolis	USA: Metropolitan Minneapolis-St Paul area
Type of study	RCT	RCT	Cluster RCT
Participants	Regular (i.e., at least 3 meals/week) customers of a university cafeteria, essentially students and university staff	Secondary schools (adolescents) and worksites (adults)	Transportation workers (*n* = 190,488)
Total study population (I/C)	209 (104/105), 156 (84/72)	Secondary school: *N* = 12, Worksite: *N* = 12	
Sex	36% male	?	79% male
Age	Mean (SD): 22.8 (3.5) years	?	19–79 years
Intervention duration	3 weeks	12 months	18 months
Follow-up			2 years
Intervention program	One portion of vegetables and two portions of fruit for free at lunchtime.	The overall design: Two kinds of setting: worksite and school Three levels of pricing: discounts of 10%, 25%, and 50% for low-fat snacksTwo levels of promotion: only label, label with sign	・Lower prices for healthy vending machine choices with 10% discount ・Low-priced fruit available from farmers’ markets held 1 day/month ・Availability (50% healthy foods; the goal of the vending machine intervention was to ensure 50% of the available vending machine offerings met healthy food criteria) ・Physical activity enhancement and fitness facilities, yoga ・Group behavioral programs (calculating calories) ・Advisory groups based at the workplace ・Self-weighing team competition
Control program	No intervention	Pricing: equal pricePromotion: no labels and no signs	Control group
ITT*	ITT analysis: 209 ⇒ 156	?	Pre-post: 78%⇔74%
Outcome	Secondary outcomes: ・Fruit (g) ・Vegetables (g) ・Energy intake (kJ) ・Energy density (kJ/100 g) ・Energy from fat (%) ・Na (mg)	Secondary outcome: Low-fat snack sale data (%)	Primary outcome: ・BMI, weight change Secondary outcomes: ・Sugar-sweetened beverages ・Fruit and vegetables (SV/day) ・Snacks, sweets (SV/day) ・Energy (kcal/day) ・Vending machine use

*Intention-to-treat test (ITT): Intervention consisting of financial incentive program versus no financial incentive program

*RCT* randomized controlled trial, *I/C* intervention/control, *SD* standard deviation, *BMI* body mass index, *SV* serving, *Na* sodium

### Results of the search

**Fig. 1:**
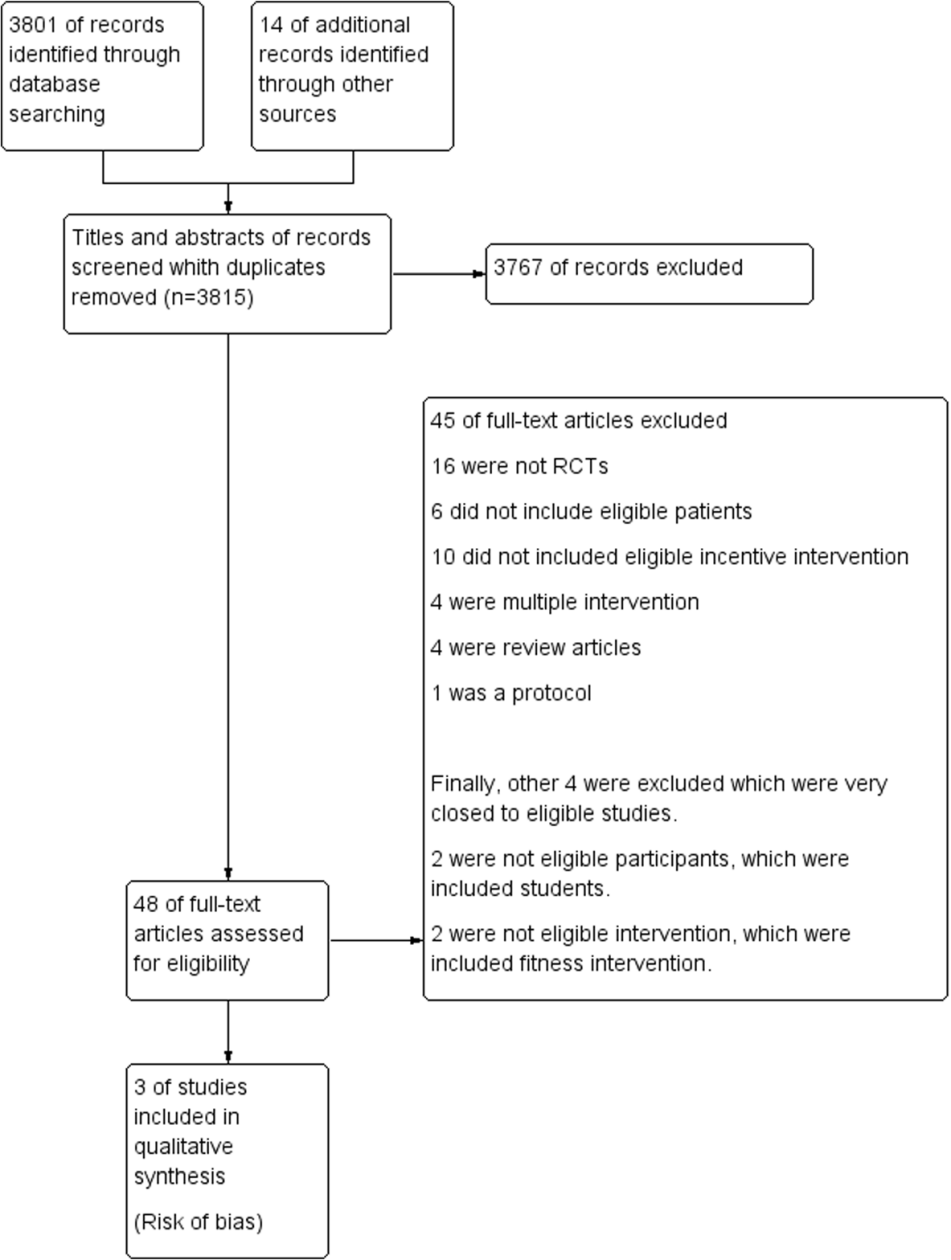
Flowchart of study selection

We screened 3815 reports from the commencement of the study to January 13, 2016, and we made an update on November 18, 2017. Of these, 48 reports were selected, and six trials from seven of these reports were found to be eligible for full-text assessment. After careful screening, we included three trials (Fig. 1) [33–35].

### Participants

The three trials comprised a total of 3013 participants, including 2059 men and 954 women who were employees aged 18 to 79 years old in various workplace settings. One trial was conducted in Philadelphia and one in Massachusetts in the USA [34, 35]; the other study was conducted in the Netherlands [33]. The American trials were conducted in a hospital [34, 35] while the Dutch trial was conducted across several settings including a hospital, industrial company, university, and police department [33].

### Interventions

All three trials focused on community-based financial incentive interventions at worksite cafeterias (the interventions did not include vending machines or kiosks). No intervention programs used social marketing. One trial [34] evaluated environmental change (food labeling) plus a price-discount intervention and small group education intervention on low-energy-density food. Another trial [33] evaluated eating behavior after exposure to the sale of smaller portion sizes with some pricing discounts. The third trial evaluated [35] reward incentive plus a feedback message as well as only a feedback message with no intervention (control). One trial assessed the impact of weight changes, BP, blood lipid levels (TC), HDL, and LDL [34]. The same trial examined the dietary intake of fruit, vegetables, bread products or dairy products, fat and sweets, and meat as well as energy intake [34]. Another intervention looked at the sales data of fried snacks [33], and the other assessed calorie intake from food consumption by sales data and food intakes by 24-h food recalls between the baseline and post-intervention [34]. The other trial used a traffic light labeling (red, yellow, green) system for the consumption of several food groups by means of register data between the baseline and post-intervention and 3-month follow-up [35].

All three trials included a financial incentive. One trial [34] combined a pricing discount (15% discount for low-energy-density or 25% discount for very low-energy-density foods among cafeteria items) with a food environment and nutritional education program. The Dutch trial included a pricing discount (35% discount for about two-thirds the size of a standard portion or 20% discount for a smaller portion size, which added to assortment and value-size pricing) and environmental interventions (food labeling and providing more healthy options) [33]. The American trial focused on group education and pricing discounts versus an environmental intervention without pricing discounts [34]. Only one trial included a reward (US$10 a month for achieving the “green goal” with all cafeteria purchases) for green-labeled items under the traffic light labeling of food groups [35]. That trial assessed consumption of green labeled items (healthy items) based on positive criteria, e.g., fruits, vegetables, or whole grains.

The Dutch trial also focused on smaller portion size with pricing discounts versus smaller portion size versus no intervention [33].

For details of the included studies, see Table 1.

## Excluded studies

We excluded 40 trials from this review (see Table 2). Of these, 16 trials did not involve randomization or their design was outside the scope of this review, six did not include eligible participants, ten had no financial incentives program, four included multiple interventions, and four were review articles.

Although four trials initially met most of our inclusion criteria for this review, we eventually excluded them because two of the studies included a combination of employees and students as participants [36, 37]. The other trials involved financial discounts and multiple interventions such as improving fitness [38, 39].

## Risk of bias in included studies

**Fig. 2:**
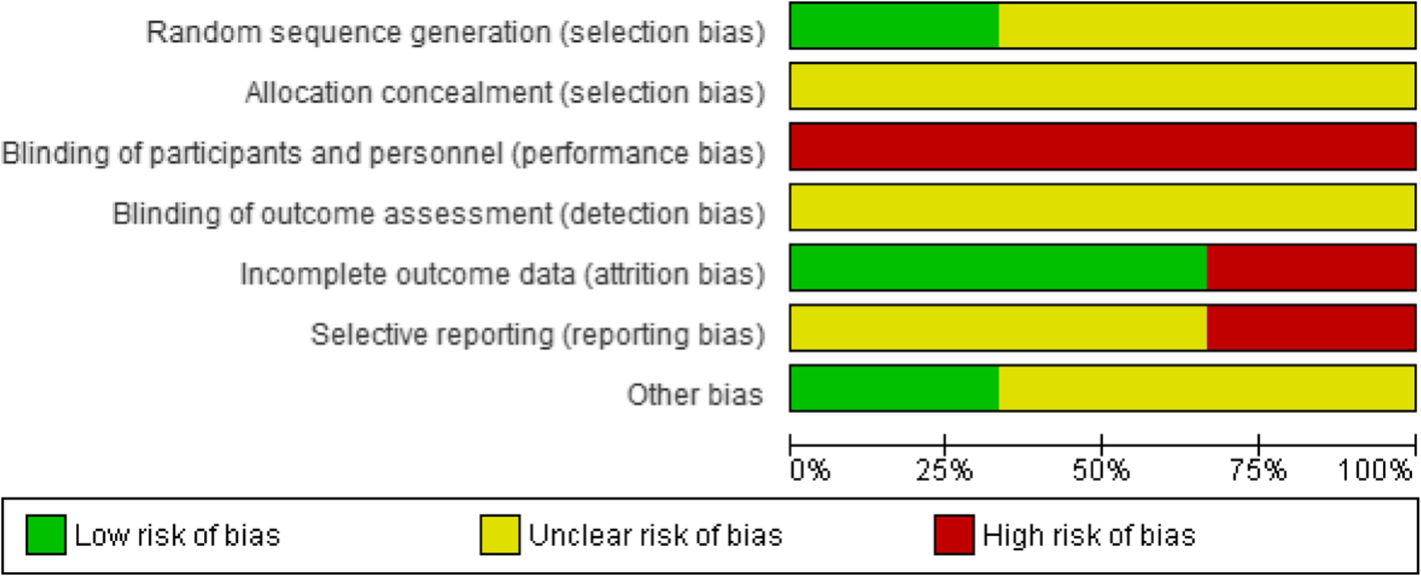
“Risk of bias” graph Review authors’ judgments about each risk of bias item presented as percentages across all included studies

**Fig. 3:**
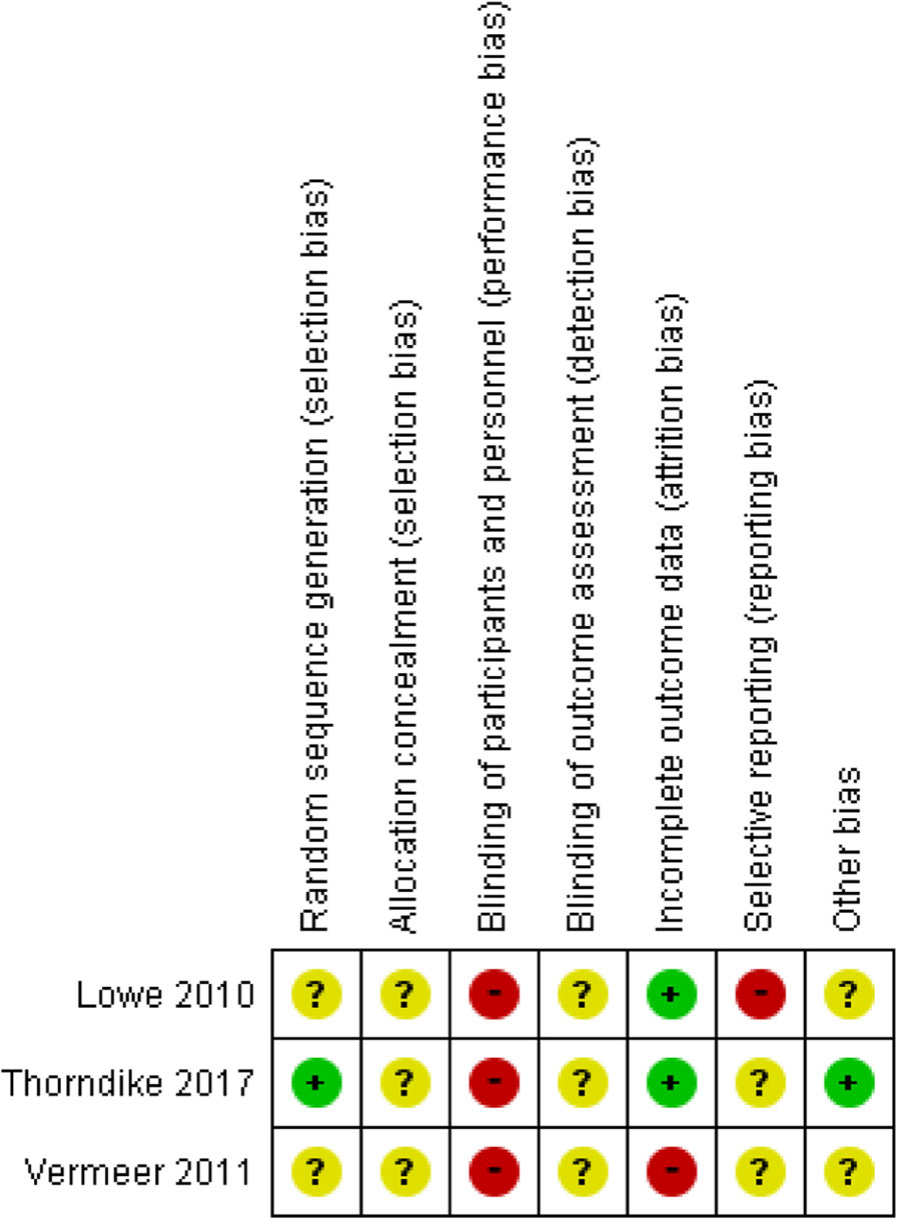
“Risk of bias” summary. Review authors’ judgments about each risk of bias item for each included study

We assessed the studies for the potential risk of selection, performance, detection, attrition, and reporting bias. See Figs. 2 and 3 for a summary of these assessments (Additional file 1).

### Allocation (selection bias)

#### Sequence generation

With two trials, risk of bias could not be adequately judged because no detailed information was provided about random sequence generation. With one trial, the sequence generation involved a simple randomization executed in Microsoft Excel (Redmond, WA).

#### Allocation concealment

The three trials provided no information about allocation concealment and were thus considered to have an unclear risk of bias.

#### Blinding (performance bias and detection bias)

In all three trials, blinding the participants to the intervention was impossible, and the outcomes might have been affected by this high risk of bias.

#### Incomplete outcome data (attrition bias)

Losses to follow-up ranged from 1.4% in the study by Thorndike et al. [35] to 44.5% in that by Vermeer et al. [33]. Two trials had a low risk of bias [34, 35] while the other had a high risk of bias [33].

#### Selective reporting (reporting bias)

One trial was as assigned a high risk of bias because no data outcomes were included in the methods [34], and two trials were assigned an unclear risk of bias because there was insufficient information for judging of low or high risk [33, 35].

#### Other potential sources of bias

Two trials had an unclear risk of bias from other sources in terms of the differences between intervention and control groups with respect to baseline characteristics were not significant for most of the reported outcomes. Only one outcome (fruit) was different at baseline but did not show any significant value [34]. One trial had insufficient information and was assigned an unclear risk of bias [33].

### Effects of interventions

See Table 3.

**Table 3:** Summary of main results

Outcome	Mean difference IV, Fixed, (95% CI)	*p* value	Effect size (*η*^2^_p_)	*p* value	Intervention Mean (SD)		No intervention Mean (SD)		No. participants (studies)	Authors	Others	Quality of evidence (GRADE)
					Pre	Post	Pre	Post				
Weight changes (kg)	0.0 (−11.69, 11.69)	1.00^*1^	0.04^*2^	0.11^*2^	85.5 (16.2)	85.9 (16.8)	78.7 (21.0)	79.1 (20.5)	96 (1)	Lowe et al.		⊕very low 1,2,3,5
Body mass index (kg/m^2^)	–		–	–	–	–	–	–	308 (1)	Vermeer et al.	Self-assessment	⊕ ⊕ low 1,2,3
HbA1c (%)	–		–	–	–	–	–	–	–	–		⊕very low 1,2,3,5
Blood pressure (mmHg)	–		–	–	–	–	–	–	–	Lowe et al.		⊕very low 1,2,3,5
Cholesterol												
Total cholesterol (mg)	16.1 (−4.67, 36.87)	0.13^*1^	0.07^*2^	< 0.05^*2^	192.4 (32.4)	201.8 (28.9)	204.1 (41.8)	197.4 (42.3)	96 (1)	Lowe et al.		⊕very low 1,2,3,5
High-density lipoprotein (mg)	4.2 (−5.66, 14.06)	0.40^*1^	0.06^*2^	< 0.05^*2^	58.4 (16.6)	60.9 (16.6)	58.7 (19.5)	57.0 (16.9)	96 (1)	Lowe et al.		⊕very low 1,2,3,5
Low-density lipoprotein (mg)	10.1 (−9.00, 29.20)	0.30^*1^	0.05^*2^	0.08^*2^	115.4 (31.6)	121.5 (31.3)	124.1 (34.4)	120.1 (37.5)	96 (1)	Lowe et al.		⊕very low 1,2,3,5
Fruit	–		0.07^*2^	< 0.05^*2^	0.77SV	0.98SV	1.41SV	0.96SV	96 (1)	Lowe et al.	24-h dietary recall	⊕ ⊕ low 1,3,4

Financial incentive intervention compared to no incentive intervention in terms of outcomes.

Patient population: workers.

Settings: workplace cafeteria.

Intervention: financial intervention (+ environmental intervention)

Comparison: no incentive intervention (+education)

*1statistically significant changes in the intervention group and no intervention group

*2repeated measures analysis reported using partial eta2 (η^2^_p_)

Effect sizes (η^2^_p_); 0.01,0.06,0.14 = small, medium, large

Quality of evidence (GRADE)

1. Random sequence generation, allocation concealment, blinding, selective reporting, and other biases high or unclear

2. Random sequence generation, allocation concealment, blinding, incomplete outcome data, selective reporting high or unclear

3. Small sample size

4. Baseline showed a significant difference for fruit consumption

5. Wide 95% CI

*SD* standard deviation, *CI* confidence interval, *HbA1c* hemoglobin A1c

### Financial incentive interventions using discounting strategies and a reward system

The three trials included financial incentive and discounts for 2059 men and 954 women aged 18 to 79 years old. For the primary outcomes, no significant effect on weight change was found in the American trial (mean difference (MD) 0.0 kg, 95% confidence interval (CI) -11.69 to 11.69, *p*=1.00 one trial, 78 women and 18 men). Using the authors’ reported effect size for repeated measures analysis with partial eta^2^ (*η*^2^_p_), weight change was *F* (1.7) = 2.56, *p* = 0.11; *η*^2^_p_ = 0.04; one trial, 78 women and 18 men aged 21 to 65 years old [34]. A cutoff of 0.01, 0.06, and 0.14 for small, medium, and large effect sizes, respectively, was used [34]. No outcomes were reported for BMI and HbA1c. For secondary outcomes, no significant effects were observed for changes in total cholesterol levels (MD 16.1, 95% -4.67 to 36.87, *p*=0.13; one trial, 78 women and 18 men) (*F* (1.66) = 5.06, *p* < 0.05, *η*^2^_p_ = 0.07; one trial, 78 women and 18 men [34]), HDL levels (MD 4.2, 95% CI -5.66 to 14.06, *p*=0.40; one trial, 78 women and 18 men) (*F* (1.66) = 4.38, *p* < 0.05, η^2^_p_ = 0.06; one trial, 78 women and 18 men [34]), LDL levels (MD 10.1, 95% CI − -9.00 to 29.20, *p*=0.30; one trial, 78 women and 18 men) (F (1.66) = 3.17, *p* = 0.08, η^2^_p_ = 0.05; one trial, 78 women and 18 men [34]), and fruit intake (F (1.71) = 5.41, p < 0.05;η^2^_p_ = 0.07); one trial, 78 women and 18 men). No outcomes were reported for BP.

As the outcomes for the intake of vegetables, fried snacks, bread or dairy products, fat and sweets, and meats, had insufficient data in the included trials, we did not calculate their mean difference and were unable to combine the data. No significant differences were found in energy intake or sales data between the incentive and no incentive groups. “Sales data” signifies cafeteria register data, which were the total unit sales at the cafeteria. With food purchases recorded at lunchtime, the approximate energy (kcal) and purchased proportion of calories from fat, protein, and carbohydrate were calculated. We were unable to analyze the mean group difference for energy intake between groups due to insufficient data.

One financial incentive involved a reward system instead of offering a discount for green-labeled items, which were positive foods (fruits or vegetables, whole grains, and lean protein or low-fat dairy as the main ingredient) [35]. The percentage of increasing green-labeled purchases was significantly greater in the incentive with feedback intervention (2.2%; *p* = 0.03) than that in the control (0.1%).

## Discussion

### Summary of main results

Incentive-based interventions with pricing strategies at workplaces provided no clear evidence of a significant reduction in the risk of body weight gain. However, such interventions may have an influence on fruit intake. Neither benefits nor harms were found for other important outcomes. This review did not integrate multiple research outcomes because the number of the RCTs was extremely small.

### Overall completeness and applicability of evidence

Incentive-focused interventions incorporating healthy menus at discounted prices in workplace cafeterias did not clearly demonstrate a significant effect in reducing body weight, but were associated with an increased intake of fruit. According to Lowe [34], fruit intake increased in the group that received incentive-based intervention involving discounted food, and decreased in the group that received no discount. This generated significant interaction between the groups indicating the effect size of increased fruit intake. However, this evidence was derived from a single study alone and was therefore included in the category of low-grade evidence. No significance was noted for the other food categories such as vegetables, or fruit and vegetables. In addition, BMI, HbA1c, and blood pressure were not verified.

In the study by Lowe et al. [34], cholesterol levels (TC, LDL, and HDL) did not show any significantly positive change. However, TC and HDL increased in the intervention group, which received a dietary environment program with additional discounting strategies (environmental change [EC]-plus group). In contrast, the group that received the dietary environment intervention alone (no discounting strategies) showed a tendency toward decreased TC and HDL levels (EC Group). This resulted in a significantly greater interaction between the two groups, which was the unexpected outcome. The participants of this research were mostly obese or overweight, with an average BMI of 29.7 (SD = 6.0). The researchers pointed out the possibility that since the average participants’ BMI was high, a backlash against energy-suppressing initiatives could occur in such individuals [34]. Furthermore, the integrity of the program itself might have been compromised because the number of menus subject to discounting strategies was small. In addition, evaluation of the intervention details from the viewpoint of TC and LDL outcomes revealed that the pricing-strategy intervention did not focus on menus with a low saturated fatty acid content, which is highly associated with LDL, but on low-energy menus.

For the present review, studies of interventions at cafeterias only were included. Studies of discounting strategies applied to vending machines or convenience stores (farmer’s markets) inside the workplace [38, 39], and incentive-based interventions providing food free-of-charge at cafeterias [36] were excluded because they were part of broader research including exercise-based intervention, or because the participants did not meet our inclusion criteria [36, 37] and might cause contamination of the intervention effect if they were included. For details of these excluded studies, see Table 2.

### Quality of the evidence

Overall, we found the methodological quality of the studies in this review to be low. We used the GRADE approach [32] to evaluate the quality of evidence of the two trials, which featured a total of 404 participants. Regarding the outcome parameters of weight change and TC, LDL, and HDL levels (all derived from one study alone) [34], we found that the sample size was small, that the assessment for risk of bias was unclear, and that the research population mostly consisted of obese overweight. The intervention group underwent a dietary environmental intervention in combination with an incentive-based intervention, whereas the comparison group underwent dietary environmental intervention alone, which meant that no pure control group was established. In both studies, the intervention group and the comparison group overlapped. In light of these facts, we determined that the assessment item “non-directness” should be rated as “serious” and concluded that the quality of the evidence was very low. For BP, we concluded that the quality of the evidence was very low due to the above-stated reasons and also because while the methodology was described, the results were not, generating a reporting bias on selective outcomes. The outcome parameter of fruit intake was rated as “not serious’” because a 24-h recording method was used for measurement; however, it was re-graded as “serious” since the conclusions given above were considered to apply with respect to the sample size and the assessment item of “non-directness.” It was ultimately determined that the quality of evidence for fruit intake was low.

### Potential biases in the review process

We strictly adhered to a protocol based on the Cochrane Systematic Review method and tried to minimize potential biases by having two reviewers independently screen and assess studies for relevance, risk of bias, and quality, and then compare their results. If the reviewers provided different results, the third and fourth authors were consulted and a consensus among all authors was reached.

### Agreements and disagreements with other studies or reviews

In the non-RCTs that were excluded from this review, positive results on purchasing rates and behavior related to pricing strategies were reported [40]. Verifying the outcomes of the physical indicators and biochemistry test results in RCTs only made us conclude that at present, we cannot clearly argue that incentive-focused interventions with pricing strategies are associated with body weight reduction. The results by An [27], who investigated purchasing behavior related to pricing strategies in their review including non-RCTs, were very similar to those obtained by this review with respect to food intake and purchasing behavior. The research [40] on pricing strategies applied to low-fat snacks sold in vending machines installed at worksites and schools demonstrated that a 10% discount did not result in a significant increase in purchasing, whereas a 25% or 50% discount significantly increased purchasing. In our included studies, one study applied discounts of 15% and 25% [34] and the other [33] gave discounts of approximately 33% by reducing the portion sizes to two-thirds. As the intensity of incentives was relatively mild due to the inclusion of the 15% discount, the research results should be carefully interpreted.

### Authors’ conclusions

#### Implications for practice

From our review, we were unable to conclude clearly the impact and effect of incentive-based interventions, such as pricing strategies, in the field: such evidence as physical indicators and biochemical test data was insufficient. However, there is a possibility that a discount-based approach at workplace cafeterias may contribute to an increased intake of fruit, which may help to prevent lifestyle-related diseases. Since the long-term effect is unknown, and these outcomes were obtained from one study in which most participants were overweight, the results may apply more to employees with a greater tendency toward obesity rather than employees in general. The three studies included in this review had an unknown or high risk of bias, and therefore the results should be carefully interpreted.

We were unable to evaluate effects of incentive programs for vending machines, shops at worksites, and other relevant places since contamination occurred with regard to inclusion criteria for participants and program details, and the number of trials and participants was small. However, the food environment approach at the workplace is important in promoting health for all employees; thus, further development of effective programs and sufficient verification are necessary.

#### Implications for research

Although the small number of studies in this review makes the effectiveness of incentive-based interventions uncertain, there is a possibility that these interventions may affect food-purchasing behavior. Higher quality RCTs are needed to investigate incentive-focused interventions using pricing strategies or any other relevant approaches with respect to body weight, BMI, and biochemistry test results. In addition, studies with long-term follow-up and greater sample sizes are necessary. Considering the possible benefit of increased fruit intake resulting from discounted pricing strategies, evaluating the effects on both blood pressure and potassium level, which is closely related to blood pressure, is important for future studies. Furthermore, the specific content of incentive programs, including but not limited to the size of the discount rate and effective programs, needs to be clarified. In future studies of interventions in food environment programs, physical indicators, biochemistry data, other relevant parameters, and study design should also be carefully evaluated to reduce the risks of bias affecting research quality and to understand correctly the impact of pricing strategies on incentive-focused interventions, for example, forming a control group in which educational intervention is not performed.

**Table:** Box 1 The PICOS criteria

Participants	Employees at any worksite, including both men and women
Intervention	Organizational-based, food-based incentive-pricing strategies or social marketing in workplace cafeterias, vending machines, and kiosks
Comparison	Any other treatment, other interventions, or placebo
Outcome	Primary outcomes (continuous variables):
	1. Changes in weight (kg)
	2. Body mass index (BMI) (kg/m2)
	3. Changes in HbA1c (%)
	Secondary outcomes:
	1. Blood pressure (mmHg)
	2. Changes in cholesterol levels (mg)
	3. Food consumption (changes in consumption of vegetables [g or serving (SV)], fruit [g or SV], fruit and vegetables [g or SV], sugary beverages [g], sweets [g], and other foods)
	4. Nutritional intake (changes in fat and oil intake [g])
	5. Changes in fiber intake (g)
	6. Changes in energy intake (kcal)
Setting	Worksite

## Additional file

Additional file 1: Risk of bias table. Risk of bias assessment for randomized controlled trials based on the Cochrane Collaboration’s risk-of-bias criteria (DOCX 22 kb)

## Abbreviations

BMI: Body mass index
BP: Blood pressure
CI: Confidence interval
EC: Environmental change
GRADE: Grades of Recommendation, Assessment, Development and Evaluation Working Group
HbA1c: Hemoglobin A1c
HDL: High-density lipoprotein
LDL: Low-density lipoprotein
MD: Mean difference
OECD: Organisation for Economic Co-operation and Development
RCT: Randomized control trial
SV: Serving
TC: Total cholesterol
US: United States
WHO: World Health Organization
