# Supplementary material for: Social marketing including financial incentive programs at worksite cafeterias for preventing obesity: a systematic review
Source: Syst Rev. 2019 Feb 28;8:66. doi: 10.1186/s13643-019-0965-0 (PMC6394016; doi:10.1186/s13643-019-0965-0)
Supplement: Supplementary file 1 — Risk of bias table. Risk of bias assessment for randomized controlled trials based on the Cochrane Collaboration’s risk-of-bias criteria (DOCX 22 kb) [file 13643_2019_965_MOESM1_ESM.docx]

| **Included studies (RCTs)** | | | |  |
| --- | --- | --- | --- | --- |
|  | **Author (years)** | **Lowe 2010** |  | |
|  | **Type of study** | RCT | |  |
|  | **Domain** | **Authors’ judgment** | **Support for judgment** |  |
|  | Random sequence generation (selection bias) | Unclear | Method of sequence generation not mentioned |  |
|  | Allocation concealment  (selection bias) | Unclear | “Participants were randomly assigned into one of two intervention groups”. Also, “randomization of participants occurred within each worksite”. However, the method of allocation concealment was not described. |  |
|  | Blinding of participants and personnel (performance bias) | High | Not possible |  |
|  | Blinding of outcomes assessment (detection bias, self-reported outcomes) | Unclear | Not mentioned |  |
|  | Incomplete outcome data (attrition bias) | Low | Total attrition rate was 19.8% at post-intervention.  However, more participants from Hospital B dropped out than from Hospital A. |  |
|  | Selective reporting (reporting bias) | High | Only blood pressure outcomes in methods were not reported. Other specified outcomes in methods were reported. Also it was unclear if a protocol of this trial had been published.  Study protocol not available. |  |
|  | Other bias | Unclear | There were no statistically significant differences in sex, ethnicity, weight, BMI, or blood lipids. There was an imbalance of baseline characteristics between groups for fruit intake, but significance was not shown. |  |
|  | **Author (years)** | **Vermeer**  **2011** | |  |
|  | **Type of study** | Cluster RCT 3-arm parallel group trial | |  |
|  | **Domain** | **Authors’ judgment** | **Support for judgment** |  |
|  | Random sequence generation (selection bias) | Unclear | Method of sequence generation not reported. |  |
|  | Allocation concealment  (selection bias) | Unclear | “The 25 remaining worksites were randomly allocated to either”; however, method of allocation concealment was not reported. |  |
|  | Blinding of participants and personnel (performance bias) | High | Not possible |  |
|  | Blinding of outcomes assessment (detection bias, self-reported outcomes) | Unclear | Not mentioned |  |
|  | Incomplete outcome data (attrition bias) | High | Total attrition rates: Intervention 1- 29.9%; Intervention 2- 44.4%; Intervention 3-42.2%. |  |
|  | Selective reporting (reporting bias) | Unclear | Study protocol not available |  |
|  | Other bias | Unclear | The study was unclear about significant differences between the intervention and control groups at baseline. No significant differences in relevant background variables were found between the participants in the final sample and the participants who only completed questionnaire 1. |  |

**Additional file: Risk of bias table**

Risk of bias assessment for randomized controlled trials based on the Cochrane Collaboration’s risk-of-bias criteria

| **Included studies (RCTs)** | | | |  |
| --- | --- | --- | --- | --- |
|  | **Author (years)** | **Thorndike**  **2016** |  | |
|  | **Type of study** | RCT | |  |
|  | **Domain** | **Authors’ judgment** | **Support for judgment** |  |
|  | Random sequence generation (selection bias) | Low | The sequence generation involved a simple randomization executed in Microsoft Excel (Redmond, WA). |  |
|  | Allocation concealment  (selection bias) | Unclear | The method of allocation concealment was not described. |  |
|  | Blinding of participants and personnel (performance bias) | High | Not possible |  |
|  | Blinding of outcomes assessment (detection bias, self-reported outcomes) | Unclear | Not mentioned |  |
|  | Incomplete outcome data (attrition bias) | Low | Attrition rate was 2.7%, 2.9%, 1.4% at post-intervention. |  |
|  | Selective reporting (reporting bias) | Unclear | Study protocol not available. |  |
|  | Other bias | Low | There were no statistically significant differences in food consumption at baseline. |  |
